# Urinary Tract Infections Caused by *K. pneumoniae* in Kidney Transplant Recipients – Epidemiology, Virulence and Antibiotic Resistance

**DOI:** 10.3389/fcimb.2022.861374

**Published:** 2022-04-21

**Authors:** Beata Krawczyk, Magdalena Wysocka, Michał Michalik, Justyna Gołębiewska

**Affiliations:** ^1^ Department of Molecular Biotechnology and Microbiology, Faculty of Chemistry, Gdańsk University of Technology, Gdańsk, Poland; ^2^ Medical Center MML, Warsaw, Poland; ^3^ Department of Nephrology, Transplantology and Internal Medicine, Medical University of Gdańsk, Gdańsk, Poland

**Keywords:** kidney transplant recipient, UTI, *Klebsiella pneumoniae*, MDR, virulence factors, asymptomatic bacteriuria, recurrent UTI, colonization

## Abstract

Urinary tract infections are the most common complication in kidney transplant recipients, possibly resulting in the deterioration of a long-term kidney allograft function and an increased risk of recipient’s death. *K. pneumoniae* has emerged as one of the most prevalent etiologic agents in the context of recurrent urinary tract infections, especially with multidrug resistant strains. This paper discusses the epidemiology and risk factors associated with urinary tract infections in kidney transplant recipients, multi-drug resistance of *K. pneumoniae* (ESBL, KPC, NDM), treatment and pathogenesis of *K. pneumoniae* infections, and possible causes of recurrent UTIs. It also addresses the issue of colonization/becoming a carrier of *K. pneumoniae* in the gastrointestinal tract and asymptomatic bacteriuria in relation to a symptomatic UTI development and epidemiology.

## Introduction

Kidney transplantation (KTx) is the renal replacement therapy of choice for a substantial number of patients with end-stage renal disease (ESRD). However, as successful KTx requires the use of immunosuppression, infectious complications are very common. Urinary tract infections (UTIs) in renal transplant recipients may deteriorate graft function and affect patient survival (Alangaden et al., 2006; Pourmand et al., 2007; Veroux et al., 2008). According to the guidelines of the Infectious Diseases Society of America (IDSA) (Nicolle et al., 2005) and European Association of Urology (EAU), UTI is defined as the presence of bacteriuria with signs of infection that may manifest with mild symptoms as an inflammation of the lower urinary tract or acute graft pyelonephritis (AGPN) (Singh et al., 2016a; Parasuraman et al., 2013; Goldman and Julian, 2019). UTIs may lead to bloodstream infections (BSI) or even to urosepsis (Li et al., 2014; Dale et al., 2018; Gołębiewska et al., 2019). Any symptomatic UTI in a transplant recipient is considered complicated, regardless of whether it affects the lower or upper urinary tract, as immunosuppression increases both the risk of infection and/or treatment failure (Parasuraman et al., 2013; Goldman and Julian, 2019; Gołębiewska et al., 2019). Although UTIs may occur at any time after transplantation, the highest incidence is reported in the first year, particularly within the first 3-6 months after organ transplantation (Abbott et al., 2004; Säemann and Hörl, 2008). In some KTx recipients UTIs tend to recur. Recurrent UTI is defined as three or more episodes of symptomatic urinary tract infections within 12 months or two episodes within last 6 months. UTIs may also present as asymptomatic bacteriuria (ABU), which is diagnosed when patient’s urine contains over 10^5^ CFU/mL bacteria, in the absence of any symptoms associated with the infection. In addition, ABU is defined as the presence of <10^5^ CFU/mL of a single bacterial species during antibiotic therapy or at least 10^2^ CFU/mL in urine collected post-catheterization (Nicolle et al., 2005; Gołębiewska et al., 2011). **Table 1** shows diagnostic criteria of UTI in renal transplant recipients.

**Table 1:** Diagnostic criteria of UTI in renal transplant recipients (Fishman and Issa, 2010; Parasuraman et al., 2013; Singh et. al., 2016a; Goldman and Julian, 2019).

Classification of UTI	Clinical presentation	Laboratory tests*
Asymptomatic bacteriuria (ABU)	No local or systemic signs of UTI	>10 WBC/mm^3^; ≥10^5^ CFU/mL in two consecutive urine samples collected after >24 h in women;≥10^5^ CFU/mL in a single urine sample in men;≥10^5^ CFU/mL in patients undergoing antibiotic treatment;≥10^2^ CFU/mL in a single urine sample collected post-catheterization
Uncomplicated UTI	Dysuria, frequent or urgent urination (normal function of the urinary tract)	>10^5^ CFU/mL in a single urine sample
Complicated UTI	Fever, pain in the region of the transplant kidney, chills, malaise or bacteraemia with the same microorganism in urine and blood cultures. Renal impairment.	≥10 WBC/mm^3^; >10^5^ CFU/mL in women; ≥10^4^CFU/mL in men or in a single urine sample collected post-catheterization in women
Recurrent UTI	At least three episodes of symptomatic UTI within 12 months or two episodes within last 6 months; it may present as episodes without structural/functional changes to the kidney.	>10^3^ CFU/mL

*WBC, white blood cells; CFU, colony-forming unit.

There is a significant difference between the incidence of UTIs among KTx recipients as compared with other solid organ transplant recipients. One cohort study (Vidal et al., 2012) followed up 2,405 recipients of solid organs for 3 years. The incidence of UTIs per 100 subjects per year was the highest for renal transplant recipients (13.84), followed by liver (3.09), heart (2.41) and lung (1.36) transplant recipients. In a retrospective analysis of the infection incidence in solid organ transplant patients who presented to the emergency department, KTx recipients were admitted over 3 times more often as compared to other patients. UTIs were the most common cause of hospitalization, accounting for 43% of cases (Trzeciak et al., 2004). The most common etiological factors of UTI in KTx recipients are similar to those identified in general population with complicated UTI. Gram negative bacteria are the leading cause of even 90% UTIs following kidney transplantation (Valera et al., 2006). *Klebsiella* sp. is the third most common bacterial species reported in this group of patients (Gołębiewska et al., 2019; Zalewska-Piątek et al., 2019; Wysocka et al., 2021). *E. coli* often causes UTIs both in KTx recipients and non-transplant patients, and *Klebsiella pneumoniae* more frequently colonizes patients who underwent renal transplantation. The relative prevalence of one bacterial species compared to the other tends to vary with the time of UTI onset after transplantation. In this review, we analyze the epidemiology of *K. pneumoniae* in KTx recipients, virulence, antibiotic resistance and the problem of antibacterial treatment in the case of *K. pneumoniae* infections.

## Epidemiology of *K. pneumoniae* UTI in Kidney Transplant Recipients

The incidence of UTIs caused by *K. pneumoniae* is remarkably diverse, ranging from 5% to 53% (Cepeda et al., 2005; Alangaden et al., 2006; Giullian et al., 2010; Fiorante et al., 2011; Vidal et al., 2012; Lee et al., 2013; Silva et al., 2013; Wu et al., 2013; Gozdowska et al., 2016; Al Midani et al., 2018; Sui et al., 2018; Singh et al., 2016b). In studies performed in Europe and Asia, *K. pneumoniae* was the second most common microorganism causing UTIs in this group of patients, following *Escherichia coli* (Wu et al., 2013; Tekkarışmaz et al., 2020; Shimizu et al., 2021; Singh et al., 2016b). In turn, *K. pneumoniae* ranked third or fourth following *E. coli, Pseudomonas aeruginosa* and *Enterococcus* spp. in all of the US studies collected (Chuang et al., 2005; Alangaden et al., 2006; Ariza-Heredia et al., 2014; Sui et al., 2018). The frequency with which *K. pneumoniae* is isolated from the urinary tract of renal transplant recipients with signs of UTI may vary at the same transplant centre at different times and at different transplant centres in a given country. This may be associated with different perioperative prevention strategies, diagnostic procedures (Kawecki et al., 2011; Adamska et al., 2015) and new immunosuppression regimens (Wu et al., 2013).

Statistical data on uropathogens causing UTIs in kidney transplant recipients presented by four groups of Polish authors showed different results depending on the transplant centre in Poland (Gołębiewska et al., 2011; Adamska et al., 2015; Gozdowska et al., 2016; Rostkowska et al., 2020). Furthermore, the results from the same centre but from different study periods also varied. Gołębiewska et al. (2011) demonstrated that *Enterococcus faecium* and *E. coli* were the predominant bacteria causing UTIs in renal transplant (RTx) patients at the Medical University of Gdańsk in 2009, with *K. pneumoniae* being third (24.5%). Adamska et al. (2015) showed that *K. pneumoniae* was the second most commonly isolated pathogen causing UTIs in this group of patients (12%) in one Clinical Hospital in Poznań in 2013-2014. In turn, Gozdowska et al. (2016) isolated *K. pneumoniae* in 15% of cases at the Medical University of Warsaw in 2013-2014, whereas Rostkowska et al. (2020) identified these bacteria in 33% of cases at the case centre in 2011-2018. This suggests that *K. pneumoniae*, being the second or third most commonly isolated bacterium, is becoming the prevailing etiological factor of UTIs at transplant centres. Changes in aetiological factors of UTI over time were also observed by Origüen et al. (2016), who compared two groups of renal transplant recipients whose procedure was performed in 2002–2004 (A) and 2011–2013 (B). Both groups were followed up for 2-3 years after treatment. In this study the number of isolated *K. pneumoniae* strains increased from 9.5% in group A (earlier) to 15.6% in group B (later). *E. coli* remained the most common single pathogen in both cohorts, but its rate in overall prevalence decreased (A — 59.5% vs. B — 46.5%). *K. pneumoniae* was shown to be the second most common pathogen in studies conducted at two sites in Turkey (Rivera-Sanchez et al., 2010; Tekkarışmaz et al., 2020), but it was also demonstrated that its rate increased from 16.9% to 23.8% over time. The highest incidence of UTI caused by *Klebsiella* spp. (46%) was noted at the transplant unit in Portugal. *Klebsiella* spp. UTI recurred post-RTx in 72% of cases at the same unit (Silva et al., 2013).

**Figure 1:**
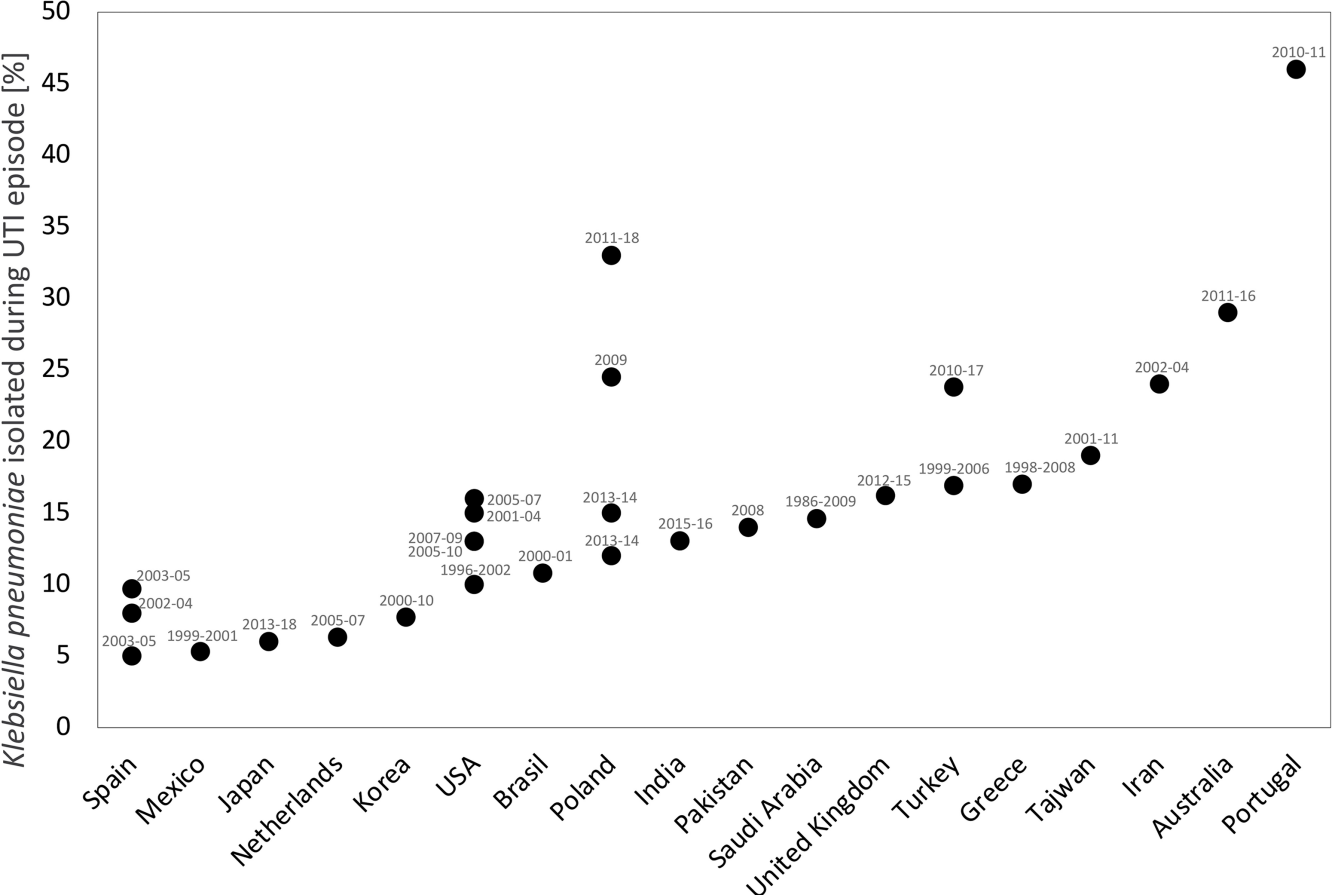
The incidence of *K. pneumoniae* UTIs in renal transplant recipients by countries in which the study was conducted. The study period (in years) is shown in the labels. This analysis was performed based on following references: Abbott et al., 2004; Charfeddine et al., 2002; Chuang et al., 2005; Alangaden et al., 2006; Dantas et al., 2006; Valera et al., 2006; Dupont et al., 2007; Memikoğlu et al., 2007; Pourmand et al., 2007; López-Medrano et al., 2008; Giullian et al., 2010; Iqbal et al., 2010; Rivera-Sanchez et al., 2010; Fiorante et al., 2011; Gołębiewska et al., 2011; Papasotiriou et al., 2011; Barbouch et al., 2012; Vidal et al., 2012; Lee et al., 2013; Lim et al., 2013; Silva et al., 2013; Wu et al., 2013; Ariza-Heredia et al., 2014; Adamska et al., 2015; Gozdowska et al., 2016; Singh et al., 2016b; Al Midani et al., 2018; Mukherjee et al., 2018; Olenski et al., 2019; Tekkarışmaz et al., 2020; Shimizu et al., 2021.

Linares et al. (2010) analysed infections caused by *K. pneumoniae* in over 1000 solid organ transplant recipients. Infections of this etiology were most common among KTx recipients and urinary tract was most frequent infection site. Extended-spectrum beta-lactamases producing strains (ESBL+ strains) caused 64% of infections diagnosed in KTx recipients, and nearly 30% of them were accompanied by bacteraemia. The majority of episodes were diagnosed shortly after transplantation. **Figure 1** illustrates the incidence of *K. pneumoniae* UTIs in KTx recipients by countries that collect statistical data.

## Pathogenesis of *K. pneumoniae* UTIs and Virulence

*K. pneumoniae* exhibits numerous different strategies to adapt to its ecological niche and protect itself against host immune response. Bacterial virulence factors play a role in the pathogenesis of UTI. Type 1 and 3 fimbriae, iron uptake system (siderophores), lipopolysaccharide (LPS), polysaccharide shell are some of important virulence factors that increase bacterial survival and disease induction (Schembri et al., 2005; El Fertas-Aissani et al., 2013; Holt et al., 2015; Paczosa and Mecsas, 2016; Martin et al., 2018; Guo et al., 2012). The pathogenesis of UTI is most frequently associated with uropathogenic bacteria in the intestine that enter the bladder through the urethra. To do so, bacteria use adhesins that help them adhere to bladder epithelial cells (Bien et al., 2012). Particular cell structures, such as adhesins, pili (fimbriae) on the surface of uropathogenic bacteria, interact with the corresponding receptors of bladder epithelial cells (Bien et al., 2012). Fimbrial adhesins are rigid protein structures that make *Klebsiella* bacilli adhere in place in the face of mechanical stress associated with an irregular urine flow in the urethra. Type 1 fimbriae and type 3 fimbriae are most important in the first stage of *Klebsiella pneumoniae* infection (Struve et al., 2009; Paczosa and Mecsas, 2016). Expression of type 1 fimbriae in *K. pneumoniae* is dependent on an invertible DNA element, named “*fim* switch”. During colonization and infection “*fim* switch” can change the orientation either to the “on” position, leading to a fimbriated state or an “off” position providing to the nonfimbriated state of bacteria (Struve et al., 2008). *K. pneumoniae* uses environmental stimuli to regulate expression of type 1 fimbriae. For example, type 1 fimbriae genes are expressed in the urinary tract but not in the gastrointestinal tract or lungs (Schembri et al., 2005; Struve et al., 2009). In addition to their primary function to mediate adhesion to host epithelial cells, type 1 fimbriae play an important role in invading the bladder cells and may be involved in biofilm formation in the bladder and on abiotic surfaces (Schembri et al., 2005; Schroll et al., 2010; Struve et al., 2008; Rosen et al., 2008). Biofilms are settled communities of microorganisms whose cells are embedded in the matrix of extracellular polymeric substances (EPS). Biofilms protect from the host immune responses defense, facilitate the cells’ survival, provide increased availability of nutrients, and better opportunities for cellular communication and exchange of genetic material. Biofilms are responsible for the ineffectiveness of antibiotics treatment blocking their access to the cells lying in the deeper layers of the biofilm matrix and generating drug resistance (Donlan, 2001; Rahdar et al., 2019). Another role of type 1 fimbriae in pathogenesis is also known. They have been found to enhance lectinophagocytosis by macrophages and neutrophils *in vivo*. Furthermore, a FimH subunit makes it easier for bacteria to bind to immune cells such as mast cells, which then leads to increased activation of immune cells and neutrophil recruitment. This may result in an increased clearance of *K. pneumoniae* (Struve et al., 2010; Ramirez et al., 2012; Paczosa and Mecsas, 2016).

Type 3 fimbriae are also secreted through the chaperone-usher system and encoded by the *mrk* genes*. mrkD* encodes the adhesin, which binds to receptors or peptides accessible from previous cell damage. Type 3 fimbriae have been found to be necessary for the formation of *K. pneumoniae* biofilm, binding to the extracellular matrix of living tissue and binding to abiotic surfaces, e.g. catheters, implants, covered with host tissue. In a study of 209 multidrug resistant bacterial strains isolated from rectal swabs taken in the first 48 hours before or after solid organ transplantation (the study population consisted mostly of KT recipients) 73% of *K. pneumoniae* strains showed moderate or strong biofilm production, in comparison to only 16% of *E. coli* strains (Ramos-Vivas et al., 2019). Presence of type 3 fimbriae in bacteria has been shown to stimulate neutrophils to produce reactive oxygen species (ROS) against which bacteria must defend themselves (Sebghati et al., 1998; Schroll et al., 2010; Struve et al., 2009).

Besides type 3 fimbriae, polysaccharide capsules encoded by the *cps* gene cluster are also involved in biofilm formation. The capsules participate in the first stage of adhesion during biofilm formation and protect against the host immune system (phagocytosis, complement) to finally form a mature biofilm structure. The fimbrial adhesin KPN, encoded by the *kpn* gene, and the outer membrane lipoprotein encoded by the *ycfm* gene also play a role in adhesion and forming a biofilm, similarly the factor *oxy*R, that protects against oxidative stress and enhances expression of type 1 and 3 fimbriae (El Fertas-Aissani et al., 2013; Vuotto et al., 2017).

After entering the bladder, bacteria may proliferate and cause bladder infection or migrate up the ureter and cause AGPN. Recurrent UTI may present as an independent urinary tract infection, persistent foreign body (i.e. a stent or drain) colonization, infection of an organ or tissue (i.e. prostatitis, pyelonephritis or abscess). However, the model described above does not satisfactorily explain numerous recurrent UTIs, in which bacterial strains causing both the initial infection and the recurrence are genetically identical. Recurrent UTIs are presumed to involve the formation of intracellular bacterial communities (IBCs), and these phenomena have been described as a type of intracellular biofilm (Anderson et al., 2003). For example, uropathogenic strains may enter the cytosol of cells lining the bladder surface and proliferate rapidly, forming a biofilm-like complex with large intracellular aggregates containing up to 10^6^ bacteria each (Anderson et al., 2003; Hunstad et al., 2010). Duraiswamy et al. (2018) demonstrated that 6 h after infection, during early IBCs formation, these aggregates contain roughly 10^3^ variable bacteria IBC formation may increase the capacity of these bacteria for colonizing the urinary tract and avoiding being flushed out by the stream of urine, the influx of inflammatory cells and contact with antibiotics. In the intermediate and basal cells of the bladder, bacteria may also enter a dormant state, becoming an inactive/resting intracellular reservoir that may periodically become active and cause UTIs (Rosen et al., 2008; Barber et al., 2013). The capacity of *K. pneumoniae* for surviving and proliferating in tissues and organs is determined by presence of appropriate ions and nutrient substrates, with iron being essential for the synthesis of cytochromes, ribonucleotide reductase and other enzymes. Therefore, survival of *Klebsiella* depends on siderophores in an environment with minimum quantities of free or unbound iron (Holden and Bachman, 2015). Siderophores are molecules that show high affinity for Fe^3+^ ions. The quantity of free iron in urine is relatively high (1-3 μg under normal conditions) (van Swelm et al., 2020). All *Klebsiella* spp. produce enterobactin. The enterobactin synthesis gene (*ent*) is a component of the core genome. Several other acquired loci associated with siderophore synthesis (aerobactin, salmochelin and yersiniabactin) are identified as common accessory genes. A bacteria may acquire them through a horizontal gene transfer (Holt et al., 2015; Paczosa and Mecsas, 2016; Choby et al., 2020; van Swelm et al., 2020). The presence of siderophores associated with genes such as *ybtS, entB, irp2, fyuA* and *iutA* may make it easier for the bacteria to colonise and survive in the patient’s body, ultimately exacerbating the infection (Holt et al., 2015). Yersiniabactin is found in approx. 30% of *K. pneumoniae* that cause UTIs (Holt et al., 2015; Lam et al., 2018a). Aerobactin and salmochelin are much less common (<5% of isolates), are encoded by the *iuc* and *iro* loci, respectively, and are usually transferred on a virulence plasmid (Wyres et al., 2019; Lam et al., 2018b; Chen et al., 2004). Occasionally, salmochelin may be found encoded on the same integrative conjugate as yersiniabactin along with colibactin, which is a genotoxin (Lam et al., 2018b). Furthermore, many recent studies report a high incidence of aerobactin in hvKp strains, suggesting that it is a critical virulence factor in hvKp (Russo et al., 2014; Choby et al., 2020). All these acquired siderophores are associated with virulence and invasive diseases, often ending with patient’s death (Rosen et al., 2008; Holt et al., 2015; Martin et al., 2018).

LPS is commonly believed to be a key virulence factor, helping *K. pneumoniae* survive in the host by suppressing the complement system. High levels of LPS are involved in triggering an overreaction by the immune system (referred to as the endotoxic shock), causing severe damage to the host. Almost all bacteria in the genus *Klebsiella* have a polysaccharide capsule. They are encoded by loci of polysaccharide capsule synthesis genes (*cps* or K), and 79 capsular types (K types) have been documented (Pan et al., 2015). Some capsule types are associated with particular bacterial virulence and determine the severity of disease in given patient (Wyres et al., 2015; Wyres et al., 2016).

In addition to varied capsular types, *K. pneumoniae* may also contain additional *rmpA* and *rmpA2* genes in its genome, determining positive regulation and overexpression of the capsule, resulting in a so-called hypermucoviscosity (HM) phenotype. It is now known that the HM phenotype is associated with *rmpA* and *rmpA2* and is unrelated to the capsule type (Kawai, 2006; Walker et al., 2019; Lam et al., 2018b). The *rmpA* and *rmpA2* genes are usually located near the *iro* and *iuc* genes on the virulence plasmids KpVP-1 i KpVP-2, respectively. The *rmpA* and *iro* genes may also co-occur on the mobile integrative conjugative element (ICE), ICEKp1, whereas *rmpA2* and *iuc* are found on virulence plasmids (Lam et al., 2018b; Wysocka et al., 2020). There are only a few reports of UTIs caused by hypermucoid *K. pneumoniae* in the literature (Liu and Guo, 2019; Russo and Marr, 2019; Wysocka et al., 2020). Wysocka et al. (2020) describes *K. pneumoniae* strains isolated from KTx recipients with asymptomatic bacteriuria, which had the ability to form a hypermucoid phenotype. There are many unresolved questions about the biochemical nature and effect of the HM phenotype. Interestingly, these isolates did not have any *rmpA/rmpA2* and *magA* despite the HM phenotype. It has been hypothesised that the hypermucoid phenotype in hypervirulent *K. pneumoniae* strains may be responsible for difficulties in catheter emptying and recurrent UTIs (Shon et al., 2013). Genomic studies show how diverse the *K. pneumoniae* population is. The proposed virulence genes and the hypervirulence phenotype lead often to confusion in the determination of “hypervirulence markers”. Putative determinants of hypervirulence *K. pneumoniae* are presented in **Table 2**.

**Table 2:** Putative determinants of hypervirulence *K. pneumoniae* (hvKp) (Hsu et al., 2011; Holt et al., 2015; Wyres et al., 2016; Lam et al., 2018a; Lam et al., 2018b; Russo et al., 2018; Walker et al., 2019; Wyres et al., 2019).

Capsular specific genes
K1 and K2 capsules - highly conserved evolutionarily within individual hypervirulent clones but also in many clones with lower virulence.
Hypervirulence markers related to capsule loci: the *magA* gene (encodes the polymerase CPS of the Wzy type) and the *wag* gene (locus component K 1 (Kl1), and Kl6, Kl16, Kl54, Kl58, Kl63, Kl113).
Hypermucoviscous phenotype does not require hyperproduction of capsule.
**Clone-specific markers**
*kfu* (mediates iron (III) uptake) and *allS* (allantoin metabolism) are preserved in clonal group 23 (CG23) but not in other hypervirulent clones.
*kvgAS* is conserved in CG86, CG65, and CG25 clonal groups but it is not found in CG23.
**Genes associated with the hypermucoid phenotype**
*rmp*A - regulator of mucoidy protein A; *rmpA2* - transcriptional regulators
Hypermucoidity is not limited to hypervirulent strains, and not all hypervirulent strains are hypermucoid in a string test. The agreement between the string test and hypervirulence in clinical or animal models varies (51–98%).
Half of the recorded *rmpA* or *rmpA2* alleles contain frameshift mutations resulting from indels in the poly (G) sequence, resulting in a loss of function and a lack of hypermucoviscous phenotype
**Iron acquisition systems**
Salmochelin *iroBCDN*, aerobactin *iutA, iucABCD* and yersiniabactin *ybt* genes.
A TonB dependent receptor with approximately 71% homology to the aerobactin *iutA* receptor (detected by PCR or sequencing) is sometimes confused with aerobactin despite the lack of *iucABCD* aerobactin synthesis genes

There is a wide variety of virulence factors in strains isolated from patients with UTI. Gołębiewska et al. (2019) demonstrated a very large variation in virulence factors, when studying 61 episodes of *K. pneumoniae* UTIs in 54 kidney transplant recipients (KTx recipients) with different immunosuppression regimens. It was also shown that the choice of immunosuppression regimen seemed to influence the occurrence of different genes encoding virulence factors. The *uge, ycfM*, and *entB* genes were less common in isolates from patients receiving everolimus, basiliximab, and thymoglobulin, respectively. The *iutA* gene was, in turn, more common in strains isolated from patients taking thymoglobulin. Furthermore, strains isolated from patients taking tacrolimus showed lower virulence. It was found that there was no relationship between the profile of virulence factors and infection of particular sections of the urinary tract, as is the case for UPEC strains (Gołębiewska et al., 2019). Differences were found in the profile of virulence factors of *K. pneumoniae* involved UTIs that were isolated from KTx patients and in the control group (without KTx, with UTI). The *uge* gene prevalence was lower in renal transplant recipients taking everolimus as compared to isolates from patients not receiving mTOR inhibitors (33.3 % vs 82.8 %) (Gołębiewska et al., 2019).

## Antibiotic Resistance of *K. pneumoniae* and Treatment Options for Renal Transplant Recipients

It is extremely important to quickly initiate antibacterial treatment of symptomatic UTIs, the goal of which is to eliminate inflammation and reduce the risk of complications, such as progression to urosepsis. Preventive antibiotic therapy administered to the recipient during perioperative period may favour antibiotic-resistant pathogens (Khosravi et al., 2014). Nosocomial infections and multidrug-resistant (MDR) bacteria, *K. pneumoniae* in particular, are distinguished among predictive factors for recurrent UTIs. It is unknown why infections caused by MDR bacteria are associated with recurrent UTIs. However, both often coincide in KTx patients with urine flow alterations, such as ureteral stenosis or vesicoureteral reflux, or underlying urological abnormalities (e.g. neurogenic bladder or chronic vesicoureteral reflux) (Freire et al., 2018; Gołębiewska et al., 2019). This may be because resistance to extended spectrum cephalosporins mediated by production of extended-spectrum β-lactamases (ESBLs) co-occurs with other factors associated with increased cell invasion and expression of fimbrial adhesins. These proteins determine the capacity of *K. pneumoniae* for forming biofilm-like intracellular bacterial communities (IBC) in bladder epithelial cells, and its higher expression increases the capacity of *K. pneumoniae* for colonizing the urinary tract and forming IBCs and biofilms (Linares et al., 2007; Linares et al., 2010; Wu et al., 2013; Silva et al., 2013; Lim et al., 2013; Anastasopoulos et al., 2015). On the other hand, it may be simply the repeated exposure to antibiotics administered because of recurrent infections, that leads to selection of resistant strains, irrespectively of the combination of virulence factors. In recent years, there are numerous reports of epidemic outbreaks caused by *K. pneumoniae* strains, both ESBL+ (SHV, TEM, CTX-M) and strains producing carbapenemases (KPC) or metallo-β-lactamases (MBL). Lautenbach et al. (2001), Biehl et al. (2014) and Biehl et al. (2019) report *bla*_CTX-M_ gene to be the most common ESBL- encoding gene, both alone and in combination with other genes. Insertion sequences, transposons, integrons, gene cassettes and plasmids may transfer genes encoding resistance between bacterial cells (Tsafnat et al., 2011; Conlan et al., 2016), thereby posing high-risk dissemination of multidrug-resistant strains in kidney disease and transplantology departments. β-lactam antibiotics constitute the broadest and most diverse group of antibiotics. This is why production of different types of β-lactamases that hydrolyse penicillins, monobactams and cephalosporins (except for cephamycin) reduces the efficacy of treatment targeted at *K. pneumoniae*. Studies on ESBL-producingisolates from Australia and South America showed that class 1 integrons may play a particular role as mobilized genetic elements in the rapid evolution of antibiotic resistance among Gram-negative pathogens and that they are epidemically associated with a specific geographic area (Roy Chowdhury et al., 2011). IS26 elements were associated with Tn21-like transposon on IncL/M plasmids and they contributed significantly to the spread of multidrug-resistant (MDR) loci in Australia, with Tn1696-like transposon located on IncA/C plasmid(s) common in the South America.

Risk factors for UTIs include colonization with ESBL-producing bacteria, delayed graft function, diabetes, prior antibiotic exposure and recurrent UTIs (Chuang et al., 2005; Alangaden et al., 2006; Dantas et al., 2006; Lansang et al., 2006; Linares et al., 2007; Linares et al., 2010; Gołębiewska et al., 2014). Treatment of infections caused by ESBL-producing bacteria may become effective by initiating β-lactamase inhibitors, such as: clavulanic acid, tazobactam, sulbactam. Sensitivity to penicillins may be achieved by combining β-lactamase antibiotics and β-lactamase inhibitors (Roy Chowdhury et al., 2011). But genes encoding β-lactamases often coexist with other types of resistance, such as resistance to aminoglycosides and quinolones (Blair et al., 2015). There are reports of the *bla*_CTX-M_ gene with fluoroquinolone resistance (FQ) (Blair et al., 2015). This is mainly true in case of Mediterranean countries (Italy, Greece) with high FQ consumption and rate of resistant strains. Data collected by the ECDC (European Centre for Disease Prevention and Control. Surveillance Atlas of Infectious Diseases) reveal that as far as *K. pneumoniae* is concerned there is a high percentage of resistant strains also in Poland, which may result from a relatively frequent use of FQ in clinical practice. Carbapenems are drugs of choice in treatment of infections caused by *K. pneumoniae* strains ESBL+. With the use of broad-spectrum antibiotics, *K. pneumoniae* strains developed resistance mechanisms that also target this group of antibiotics. It was *K. pneumoniae* in which KPC-1 was identified for the first time in the U.S. (North Carolina) in 1996 (Yigit et al., 2001). According to European Centre for Disease Prevention and Control [Annual report of the European Antimicrobial Resistance Surveillance Network (EARS-Net), www.ecdc.europa.eu/en/healthtopics/antimicrobial_resistance/database (22.05.2017)] and Grundmann et al. (2017) KPC is nowadays being identified in other species of the family *Enterobacteriaceae*. The *bla*_KPC_ gene producing class A carbapenemase (serine carbapenemase) was found on Tn3-like transposons, Tn4401 (transposon) on mobile plasmids and even as an insert in the prophage sequence on bacterial chromosome (Yigit et al., 2001). Currently, *bla*_KPC-2_ and *bla*_KPC-3_, which confer resistance to penicillins, carbapenems, cephalosporins, cephamycins and monobactams, are the most common variants of carbapenemases in many countries (Yigit et al., 2001; Conlan et al., 2014; Cicora et al., 2015). Thirteen cases of nosocomial infections with *K. pneumoniae* KPC-2, ST258 in renal transplant recipients were reported in an Argentinian hospital in 2011-2013 (Chen et al., 2015). The authors concluded that carbapenems in monotherapy have a higher failure rate in KTx recipients due to increasing resistance than combination therapy based on meropenem, colistin and tigecycline (Chen et al., 2015). KPC infections may be of different origin. There are clinical reports of carbapenem-resistant *Klebsiella pneumoniae* bloodstream infections (CRKP-BSI) shortly after deceased donor renal transplant (Mathers et al., 2015; Cicora et al., 2015). The issue of donor organs infected with KPC strains is not isolated. Goldberg et al. (2012) and Mularoni et al. (2015) described cases of organ transplantation from a donor with established bacteraemia or with transplanted organ infected/colonized by multidrug-resistant *K. pneumoniae* KPC strains. In such case, organ recipients are at high risk of infection and kidney transplantation from a donor with UTI and infectious sepsis should be avoided.

Imipenemases IMP, VIM (Verona-integron-imipenemase) and New Delhi metallo-β-lactamase 1 (NDM-1) – the newest, rapidly spreading variant that poses an enormous epidemic risk in hospital setting may be distinguished among the most important acquired resistance associated with metallo-β-lactamases (MBLs). *K. pneumoniae* NDM-1 strains are dangerous because of the *bla*_NDM-1_ gene present on the mobile plasmid, which favors their spread in the hospital setting in particular; additionally, the co-occurrence of other resistance genes in its neighborhood gives the bacteria resistance to all β-lactams, fluoroquinolones and very often aminoglycosides or cotrimoxazole (Deshpande et al., 2010). Treatment options for infections with NDM-1 strains are extremely limited, which increases the mortality rate (Wang et al., 2018). Wilkowski et al. (2016) suggest prolonged combination therapy followed by oral prophylaxis with fosfomycin to treat infections caused by *K. pneumoniae* NDM-1. The use of fosfomycin has been proposed as a carbapenem-sparing strategy in antimicrobial stewardship programs. This drug is available for intravenous use as fosfomycin disodium and as fosfomycin trometamol (FT) as an oral formulation. The oral bioavailability of FT is 30% to 37% and achieves clinically relevant concentrations in the kidney, even though decreased excretion of fosfomycin in urine is observed. FT causes minor side effects, but no reports of nephrotoxicity or interactions with immunosuppressants have been reported (Surber, 2016). However, FT should not be recommended for patients with ABU (Ten Doesschate et al., 2019), and cases of resistance to fosfomycin are also reported (Karageorgopoulos et al., 2012). Wang et al. (2022) showed that resistance to fosfomycin in carbapenem-resistant *K.pneumoniae* resulted from the presence of either chromosomal *fosA* ^KP^ I91V commonly combined with *glpT* and *uhpT* transporter deficiencies or plasmid-borne *fosA3* gene.

New treatment options for the treatment of UTIs caused by carbapenem-resistant *K. pneumoniae* include ceftolozane - tazobactam, ceftazidime - avibactam, or meropenem- vaborbactam or cefiderocol. Current data suggest that these therapeutic options are promising, but their use depends on the severity of renal dysfunction, and individual dosing required for clinical efficacy, safety and tolerability (Giancola et al., 2016; Cultrera et al., 2020; Chapelle et al., 2021).

Co-administration of many antibiotics may increase pharmacodynamic activity in killing bacteria and possibly inhibit or delay development of resistance by broadening the spectrum of action and using different mechanisms of action (Levey et al., 2005; Karuthu and Blumberg, 2012; Furian and Rigotti, 2013; Conlan et al., 2014). Freire et al. (2018) claim that there are two reasons why combination therapy should be used very cautiously in transplant recipients. One of the reasons is nephrotoxicity caused by drugs used to treat *K. pneumoniae* KPC (aminoglycosides, polymyxin) and low urine concentrations of other drugs with adequate efficacy, such as tigecycline and polymyxin B (Taglietti et al., 2013). Common complications of kidney transplantation e.g. acute graft rejection, required dialysis and concomitant diseases, make it necessary to refrain from using nephrotoxic drugs that may deteriorate patient’s condition (Wilkowski et al., 2016). Rigorous monitoring of KTx recipients both during and after the infection should be considered. Such an approach makes it possible to avoid recurrences and unfavorable systemic treatments. According to Wojciechowski and Chandran (2013), a 30-day course of ciprofloxacin lower the incidence of UTI.

Fecal microbiota transplantation (FMT) from healthy donors is an unconventional solution for KTx recipients with recurrent UTIs. Grosen et al. (2019) demonstrated that FMT may be an effective way to prevent recurrent UTIs with *K. pneumoniae* ESBL+ in KTx recipients. No multidrug-resistant *K. pneumoniae* bacteria were identified in urine or feces for 12 months. It was shown that changes in the gut microbiota profile may become a strategic therapy, which makes it possible to avoid recurrent infections with carbapenem-resistant strains (Grosen et al., 2019; Ramos-Martínez et al., 2020). It seems that correction with probiotics or fecal transplantation may restore the intestinal balance in patients and protect them from UTIs. This approach reduces the use of antibiotics in treatment of patients with urinary tract disorders and resistance acquired by microorganisms.

Bacteriophage therapy can be also a potential alternative against MDR urinary tract infections (Chegini et al., 2021). Bacteriophages are viruses that attack bacterial cells causing lysis of host (lytic lifestyles) or insert into the bacterial genome, existing as a prophage (lysogenic state). Bacteriophages are isolated from various niches, e.g., soil, waters (rivers, ponds, estuaries, canals) as well as sewage (Townsend et al., 2021), and human/animal body. In the human body, they can be found in the gastrointestinal tract, faeces, saliva and the urinary tract (Keen, 2015). Hoyles et al. (2015) demonstrated the gut microbiota as a source of clinically relevant phages. They isolated a lytic phage - named KLPN1, on a strain identified as *Klebsiella pneumoniae* subsp. pneumoniae (capsular type K2, *rmpA*+). Miller-Ensminger et al. (2018) analyzed bacteriophages within the urinary microbiota to find a large diversity of previously uncharacterised phage species present in the bladder. They demonstrated differences between healthy individuals without UTIs and those with urinary symptoms; this may suggest that phages in the bladder may contribute to urinary health. The treatment of bacterial infections with phages has been known worldwide since the beginning of the 20th century (D’Herelle, 1917). Many studies report diverse lytic bacteriophages to *K. pneumonia* and their potential *in vitro* (Bogovazova et al., 1991; Kęsik-Szeloch et al., 2013; Hoyles et al., 2015). Hung et al. (2011) described experimental phage therapy in treating *Klebsiella pneumoniae*-mediated liver abscesses and bacteremia in mice (Hung et al., 2011), another experiment has indicated lytic activity of bacteriophage ZCKP1 (Myoviridae family) to *K. pneumoniae* in diabetic foot patients. (Taha et al., 2018). Phages in the urinary tract in humans are likely to play a role in local antimicrobial defence. According to Ujmajuridze et al. (2018) bacteriophage therapy might be also effective and safe for treating UTIs. Phages can be administered alone (selected doses of phages or phage cocktail) (Townsend et al., 2021) or together with antibiotics or disinfectants, observing the synergism of action for this combination (Watanabe and Watanabe, 1959). A very important feature of phages is their ability to degrade biofilms formed by the uropathogenic strains (Miller-Ensminger et al., 2018). Phages produce polysaccharide depolymerase (PDs) (Adams and Park, 1956), which are involved in the recognition and depolymerization of capsular and structural polysaccharides, including bacterial exopolysaccharides (Chibeu et al., 2012). EPS represents almost 90% of all biomass of bacterial biofilm, hence bacteriophages may be useful in the eradication of biofilm and thus antibiotic therapy is also more effective. Studies of the Polish team investigating phage therapy showed both treatment success and improvement of kidney allograft function in KTx recipients with complicated UTIs. This indicates the high potential of phage therapy in urology and nephrology, including KTx recipients (Górski et al., 2003). However, we should keep in mind, that bacteriophage therapy requires full microbiological, physicochemical, and genetic characterisation of phage, as well as knowledge of the adaptation of bacteriophage to defence systems of the host (Sulakvelidze et al., 2001; Jończyk et al., 2011; Ryan et al., 2011; Kęsik-Szeloch et al., 2013). A phage cocktail consisting of a variety of lytic phages with a short lysis period and without dangerous toxins or antimicrobial resistance genes should ensure the most effective therapy of *Klebsiella* infections (Townsend et al., 2021). So far, single case reports have been published showing successful use of phage therapy in the treatment of relapsing ESBL-producing *K. pneumoniae* UTIs in KTx recipients (Kuipers et al., 2019; Rostkowska et al., 2021).

Colonization and Being a Carrier as an Issue in Recurrent UTIs and Nosocomial Infections

The gastrointestinal tract of healthy subjects and animals and even water constitute a reservoir of *K. pneumoniae* (Blaak et al., 2015; Gorrie et al., 2017; Korach-Rechtman et al., 2020). *K. pneumoniae* colonizes the gastrointestinal tract in 35% of population – these are the so-called community-acquired (CA) strains (Ramos-Martínez et al., 2020; Korach-Rechtman et al., 2020). But in hospital setting (hospital-acquired (HA) strains) 20%-77% of patients are carrying these bacteria in their digestive systems (Aumeran et al., 2010; Gorrie et al., 2017; Decker et al., 2018). Although *K. pneumoniae* is well known to be a common cause of infections in hospitalized patients, the sources of these infections are not yet well understood. There are many possible reservoirs from which an infection may originate: employees, visitors or patients admitted to the hospital; equipment used in procedures. It is mainly the gastrointestinal tract that is the reservoir of *K. pneumoniae* and represents the risk of transmission and infection, mainly to the urinary tract and blood. Antibiotic treatment commonly used in renal transplant recipients disturbs the intestinal microbiota of patients, giving way to hospital-acquired (HA) *K. pneumoniae*. It appears that approx. 5% of colonized patients develop infection with microorganisms of the same genotype (Davis and Matsen, 1974; Martin et al., 2018; Decker et al., 2018). KTx recipients colonized with *Klebsiella pneumoniae* ESBL-producing strains were at a higher risk of developing symptomatic UTIs (Wilkowski et al., 2018), and over 60% of recurrent UTI episodes in this group of patients were caused by genetically similar strains (Espinar et al., 2015). Hands of hospital staff may also be a vector of microbial transmission (Davis and Matsen, 1974; Aumeran et al., 2010; Decker et al., 2018). In an epidemic study conducted from December 2008 to August 2009, 16 patients who underwent the same surgical procedure developed a *K. pneumoniae* infection that was later linked with to the endoscope used in the procedure (Aumeran et al., 2010). Cohort studies conducted by Jørgensen et al. (2017) of outpatients with UTI from 2009 to 2011 showed that different strains commonly produce ESBL even within the same host and microorganisms are found in feces even several months after infection. This is particularly true for catheterized or immobilized patients, or those with prior infection with ESBL strains (Brakemeier et al., 2017; Jørgensen et al., 2017). Cross-transmission of ESBL-producing strains should be taken into consideration at healthcare facilities. KTx recipients undergo various diagnostic and monitoring procedures entailing a high risk of being colonized with and becoming carriers of these strains.

Asymptomatic bacteriuria (ABU) poses a significant issue in recurrent infections and hospital epidemiology. Immunosuppressed patients may not respond with symptomatic UTI despite high urinary bacterial titres. Immunosuppressive therapy is believed to be the most common cause of ABU. Gołębiewska et al. (2019) who analyzed gene profiles encoding specific virulence factors of *K. pneumoniae* isolated from patients with ABU, revealed that the *kpn* gene encoding FimH-like adhesin prevailed in isolates in ABU patients as compared with isolates acquired from patients with symptomatic UTIs. It has been demonstrated that the *kpn* gene may replace the expression of type 1 fimbriae, facilitating bacterial colonization and persistence in patients with ABU (Gołębiewska et al., 2019). Absence of UTI symptoms may result from colonization of the urinary tract by defective strains that do not cause symptoms, but an effect of immunosuppressive treatment should also be taken into account. The intestinal microbiota of outpatients without complicated UTIs plays a protective role against urinary tract infections, and in many cases asymptomatic bacteriuria resolves spontaneously after a few days. Normal intestinal bacterial flora is capable of stimulating the production of unique antibodies in the mucosa, the so-called secretory IgA. IgA in the intestinal lumen is considered to be the first line of defense against the invasion of pathogens (Corthésy, 2013). On the other hand, microorganisms colonizing the lower urinary tract and causing ABU may pose a risk for upper urinary tract (kidneys), since the immune system is suppressed by immunosuppressive therapy and does not offer a chance for a spontaneous recovery.

A question whether antibiotics prevent asymptomatic UTIs or increase the risk of selecting antibiotic-resistant bacteria is constantly being asked. Cai and Bartoletti (2017) claim that ABU in patients with recurrent UTI (rUTI) does not require treatment because it is not an infection, and treatment is associated with emergence of antibiotic-resistant strains. Coussement et al. (2018) attempted to determine whether antibiotics used to treat asymptomatic UTIs reduced the risk of graft rejection and whether antibiotics improved kidney graft function. A study of 112 participants showed no adverse reactions to antibiotic treatment, but there was insufficient evidence to support the need for antibiotic treatment in renal transplant recipients with asymptomatic bacteriuria prior to kidney transplantation. This is why, the authors have doubts about whether to recommend a screening strategy to detect asymptomatic bacteriuria by cultures. So far there have not been no studies published dedicated solely to the topic of *Klebsiella pneumoniae* ABU. It should be noted that colonized patients carry uropathogenic *K. pneumoniae* strains. These patients are hospitalized or diagnosed at hospital units and pose an epidemic risk to other patients, especially when MDR strains are responsible for ABU.  

## Conclusion

**Figure 2:**
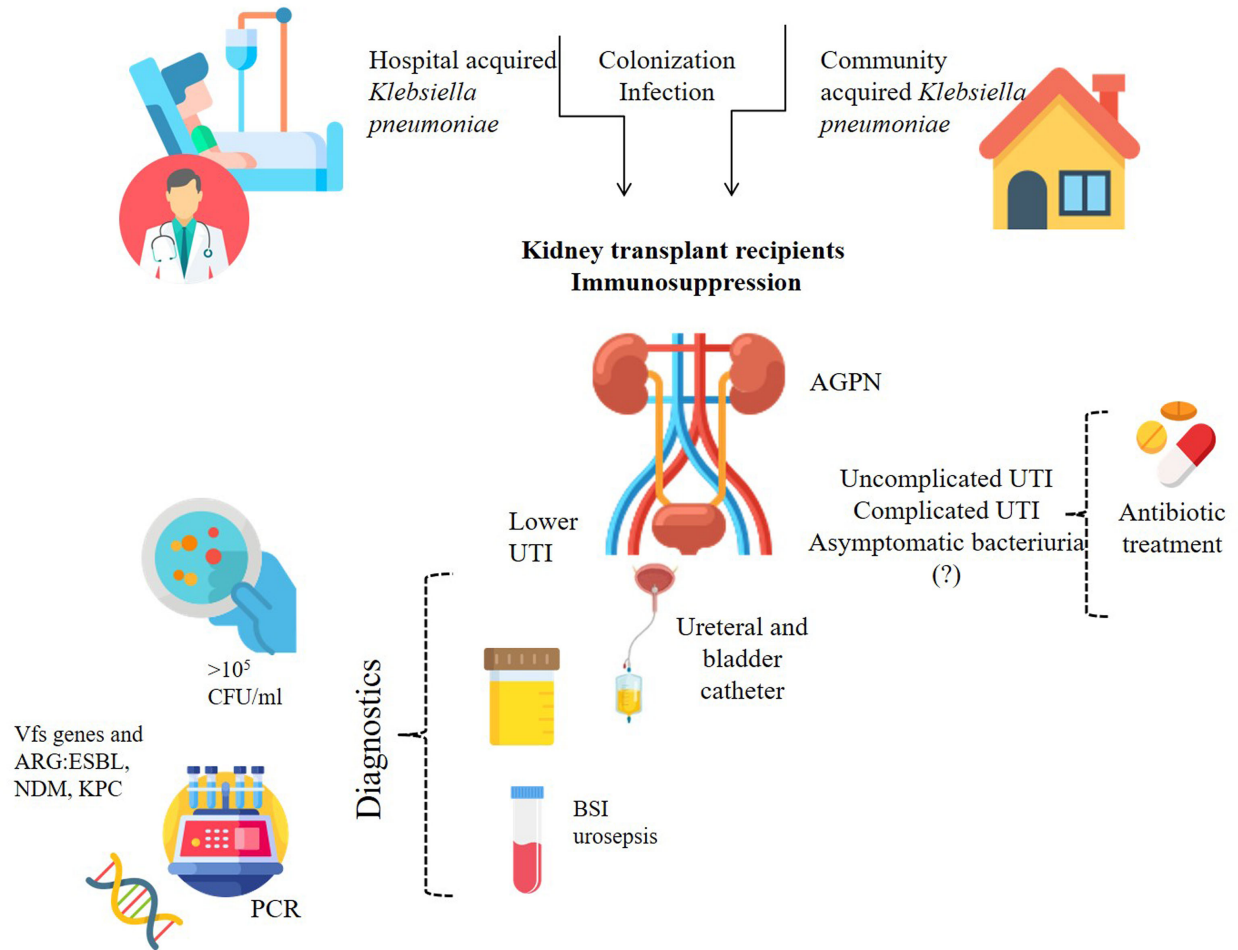
Risk factors related to *K. pneumoniae* for renal transplant recipients. BSI, blood stream infection; Vfs, virulence factors; ARG, antibiotic resistance gene/s; ESBL, Extended-spectrum β-lactamases; NDM-1, New Delhi metallo-β-lactamase 1; KPC, carbapenemases.

Kidney transplant recipients are at great risk of developing UTIs. Risk factors for *K. pneumoniae* UTIs in kidney transplant patients are shown in **Figure 2**. The etiology of UTI in these patients may differ in various transplant centers throughout one country and throughout the world, requiring a different approach to treatment. It is therefore important to collect data on the epidemiology of infections and antimicrobial susceptibility specific to a given site and population. Still, *Klebsiella pneumoniae* emerges as one of the leading UTIs causative agents worldwide, especially in case of recurrent infections with MDR strains, in KTx recipients with urine flow alterations, such as ureteral stenosis or vesicoureteral reflux, or underlying urological abnormalities (e.g. neurogenic bladder or chronic vesicoureteral reflux). Both hospital and community strains of *K. pneumoniae* can lead to complications and cause therapy problems. Patients with ABU should be treated as a possible source of multidrug-resistant *Klebsiella pneumoniae* strains, which are capable of transferring resistance and virulence genes within their population and between species in the hospital setting. Horizontal gene transfer is related not only to antibiotic resistance, but also promotes the spread of virulence among *K. pneumoniae* strains and may even lead to the emergence of hypervirulent or hypermucoid strains. Prudent use of antimicrobials is of unprecedented importance in the era of widespread antimicrobial resistance.

## Author Contributions

Conceptualization: BK, MW, JG, and MM. Collected date: MW and BK. Writing original draft preparation: MW, BK, and JG. Visualization: MW and BK. Writing—review and editing: BK. Supervision: MM and JG. All authors approved the final version of the manuscript and agreed to be accountable for all aspects of the work.

## Conflict of Interest

The authors declare that the research was conducted in the absence of any commercial or financial relationships that could be construed as a potential conflict of interest.

## Publisher’s Note

All claims expressed in this article are solely those of the authors and do not necessarily represent those of their affiliated organizations, or those of the publisher, the editors and the reviewers. Any product that may be evaluated in this article, or claim that may be made by its manufacturer, is not guaranteed or endorsed by the publisher.
